# Low temperature-induced cold shock protein modulates determinate growth in cucumber

**DOI:** 10.1186/s43897-025-00199-3

**Published:** 2026-03-01

**Authors:** Linghao Liu, Haifan Wen, Tiefeng Song, Xiangyu Wang, Junsong Pan, Jian Pan, Tianlai Li

**Affiliations:** 1 College of Horticulture, Shenyang Agricultural University, Shenyang, 110866 China; 2 School of Agriculture and Biology, Shanghai Jiao Tong University, Shanghai, 200240 China; 3 The Modern Facilities Horticultural Engineering Technology Center, Shenyang Agricultural University, Shenyang, 110866 China; 4 The Key Laboratory of Protected Horticulture, Ministry of Education, Shenyang, 110866 China; 5 School of Life Sciences, Liaoning University, Shenyang, 110036 China; 6 Liaoning Academy of Agricultural Sciences, Shenyang, 110161 China

Growth habit is a critical trait in higher flowering plants, significantly influencing crop yield and other related agronomic characteristics (Pnueli et al. 1998). The growth habits of plants are generally categorized into determinate and indeterminate types, which are determined by the state of the apical meristem. Low temperature induces the differentiation of floral organs (Périlleux et al. 2019), and cultivated cucumber, due to the domestication of the *FT* gene (Wang et al. 2019), exhibits a unique growth pattern where both vegetative and reproductive growth occur simultaneously. As a result, low temperatures during the seedling stage can easily cause the plant to form a terminal flower phenotype, leading to determinate growth and adversely affecting agricultural production. Studies have shown that determinate growth in plants is regulated by the highly conserved *TFL1* gene (Périlleux et al. 2019). In *Arabidopsis*, *TFL1* is expressed in the shoot apical meristem (SAM), where it inhibits the production of floral meristems, thereby promoting indeterminate growth in wild-type plants (Bradley et al. 1997). Overexpression of *TFL1* suppresses the expression of flowering genes, delaying the transition to reproductive growth and resulting in a delayed flowering phenotype with the formation of a multi-branched inflorescence (Bradley et al. 1997). In contrast, the apical meristem of *tfl* mutants is replaced by a floral meristem, leading to determinate growth (Shannon et al. 1991). Previous studies have shown that the CsNOT2a interacts with CsTFL1 and CsFDP to inhibit determinate growth and terminal flower formation in cucumber. CsFT (FLOWERING LOCUS T) is a key regulator promoting the transition from vegetative to reproductive growth by activating downstream flowering genes. *CsLFY* (*LEAFY*) is essential for shoot meristem maintenance and promotes flower development through interaction with CsWUS (Zhao et al. 2018; Wen et al. 2019). Further studies have shown that the TFL1-FD complex regulates target genes by competing with FT, further elucidating the complex gene regulation network controlling flowering time and plant architecture (Zhu et al. 2020). Loss-of-function of *CsTFL1* and its homolog *CsTFL1d* in cucumber results in an environment-dependent determinate growth phenotype (Wen et al. 2019, 2021), yet the molecular mechanisms underlying the environmental effects on determinate growth remain unclear.

To investigate the molecular mechanisms underlying low temperature-induced determinate growth, we planted the *S94* inbred line in early spring (February, the average temperature is 10–15 °C) and autumn (August, the average temperature is 25–35 °C). Statistical analysis revealed that the plants planted in early spring exhibited determinate growth (terminal flower formation around the 10th node), while the autumn-planted plants exhibited indeterminate growth (Fig. 1A). To investigate the molecular mechanisms underlying environmental effects on determinate growth, we performed transcriptome sequencing analysis on the plants grown in both seasons. The KEGG analysis revealed that many differentially expressed genes between the early spring (February) and autumn (August, the average temperature is 25–35 °C) groups were significantly enriched in metabolic pathways related to environmental information processing (Fig. S1). A heatmap analysis of the most differentially enriched genes is presented in Table S1. Notably, several genes involved in flower development and those associated with low temperature were both enriched in pathways linked to environmental information processing. Among the differentially expressed genes between the control and low-temperature-treated samples, one encodes a cold shock protein (CSP2) responsive to low temperature (Fig. 1B). Studies have shown that AtCSPs are involved in regulating the cold tolerance of *Arabidopsis thaliana* and play important biological functions in the growth and development processes such as seed development and silique length (Kim et al. 2007). Therefore, we selected *CsCSP2* for further research.

**Fig. 1:**
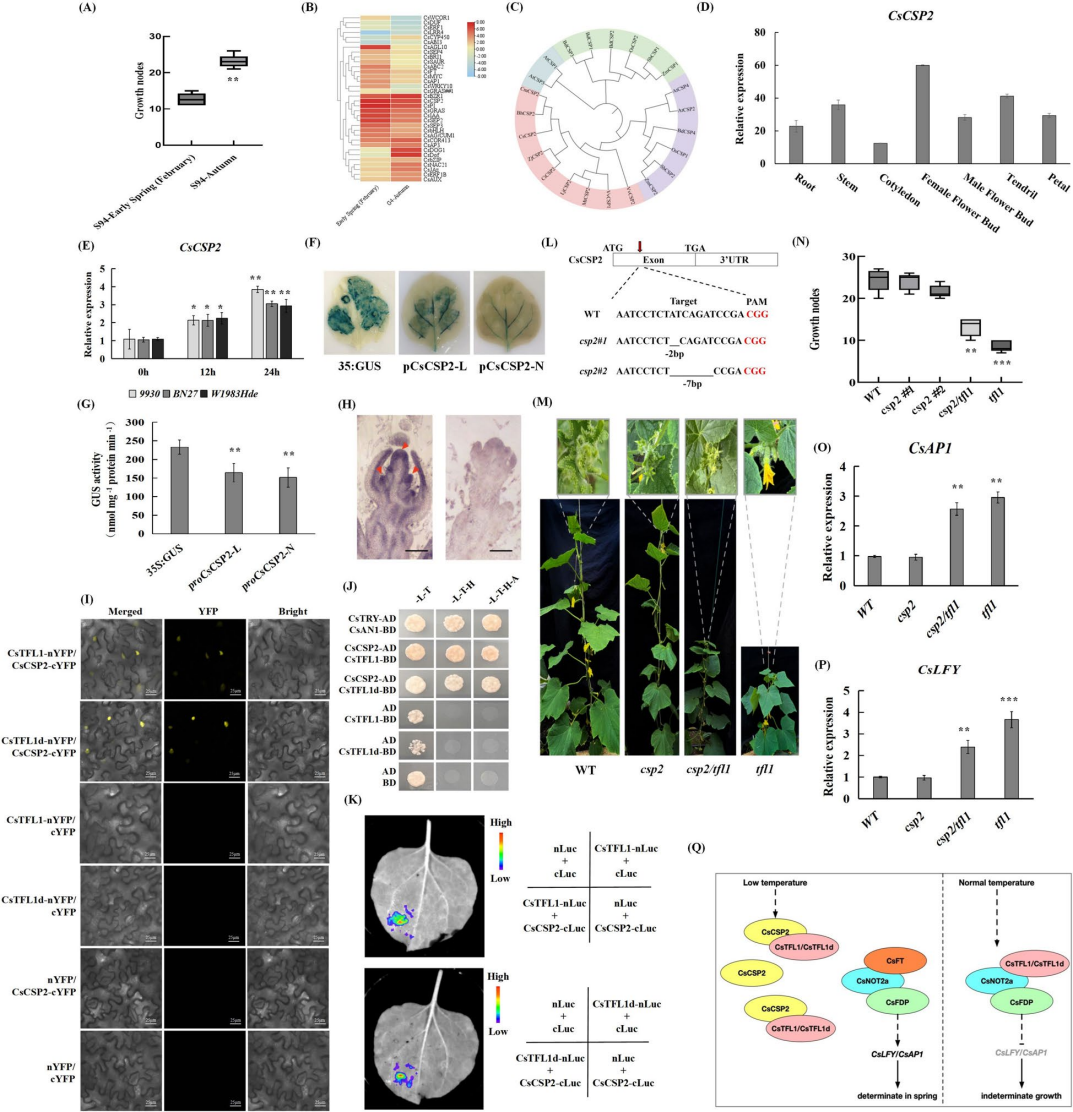
CsCSP2 responds to temperature signals and interacts with CsTFL1/CsTFL1d to regulate determinate growth in cucumber. **A** The growth nodes of the terminal flower formed in *S94* in early spring (February, the average temperature is 10–15 °C) and autumn (August, the average temperature is 25–35 °C) in 2024, respectively (*n* = 10, Student's t-test, **p < 0.01). **B** Cluster analysis of early spring (February, the average temperature is 10–15 °C) and autumn (August, the average temperature is 25–35 °C) DEGs in cucumbers. Red represents high expression level. **C** Phylogenetic analyses for CSPs protein. The trees were constructed via the neighbor-joining method with a Poisson correction model and 1000 bootstrap replicates. **D** *CsCSP2* Expression patterns in different tissue sites of cucumber. **E** Expression patterns of *CsCSP2* in cucumber of three different ecotypes under low temperature (early spring) (February, the average temperature is 10–15 °C) or normal temperature (autumn) (August, the average temperature is 25–35 °C). (*n* = 3, Student's t-test, *P < 0.05 or **P < 0.01). **F** Histochemical GUS staining in the different temperature. **G** Fluorometric GUS assays of tobacco leaves in comparison with the positive control CaMV35S promoter under different temperature. Of them, *proCsCSP2*-L stands for the low-temperature treatment at 12 °C and *proCsCSP2*-N for the room-temperature treatment at 25 °C. Error bars show standard deviation. GUS activity was measured in pmol 4 MU/μg protein/min. Values represent the mean ± standard deviation from three independent transgenic lines and each line five individual plants for each construct. (Student's t-test, **P < 0.01). **H** In situ hybridization of *CsCSP2* in gynoecy cucumber buds. Hybridization with antisenseprobe in stage 7 (left), stage 8–2 (middle). Black arrows indicatehybridization signal. Hybridization with sense-probe presents in the right. St: stamen, Pi: pistil. Bar = 200 μm. **I** BiFC assays showing the physical interaction between CsCSP2 and CsTFL1/CsTFL1d. **J** Y2H assay revealing the interaction between CsCSP2 and CsTFL1/CsTFL1d. Transformed yeast cells were grown on SD/-Trp/-Leu, SD/-Trp/-Leu/-His medium and SD/-Trp/-Leu/-His/-Ade medium. **K** LCI assays showing the physical interaction between CsCSP2 and CsTFL1/CsTFL1d in vivo. **L** Generation of *csp2* knockout lines by CRISPR‐Cas9‐mediatedgenome editing in the *S94* background. And sanger sequencing of the CRISPR edited sites in *csp2#1* and *csp2#2* mutant. **M** Phenotypes of the *csp2#1*, *csp2#2*, *csp2*/*tfl1*, *tfl1* mutant and WT planted in early spring (February, the average temperature is 10–15 °C). **N** The growth nodes of the terminal flower formed in *9930*, *csp2#1*, *csp2#2*, *csp2*/*tfl1* and *tfl1* in early spring (February, the average temperature is 10–15 °C), respectively (*n* = 10, Student's t-test, **p < 0.01). **O** The expression level of *CsAP1* in *csp2* knockout lines planted in early spring (February, the average temperature is 10–15 °C) through qRT-PCR. (*n* = 3, Student's t-test, **p < 0.01). **P** The expression level of *CsLFY* in *csp2* knockout lines planted in early spring (February, the average temperature is 10–15 °C) through qRT-PCR. (*n* = 3, Student's t-test, **p < 0.01). **Q** The proposed model of CsCSP2 and CsTFL1/CsTFL1d in modulating determinate growth of cucumbers

To investigate the evolutionary relationships of CSP proteins among various plant species, numerous homologous CSPs were obtained from GenBank. These genes were used to construct a phylogenetic tree based on the maximum likelihood method (Table S2). The analysis showed that CSP proteins can be broadly classified into four groups. Phylogenetic results showed that cucumber CsCSP2 shares a relatively close evolutionary relationship with melon CmCSP2 and wax gourd BhCSP2, based on amino acid sequence similarity (Fig. 1C). In rice and *Arabidopsis*, CSP family genes such as OsCSPs and AtCSPs exhibit broad expression across various tissues, suggesting a conserved role in plant development and stress responses (Chaikam and Karlson 2008; Kim et al. 2007). Tissue-specific expression analysis revealed that *CsCSP2* was expressed in multiple organs, with relatively higher expression levels observed in shoot apex, tendril, and flower buds, suggesting its potential role in meristem activity and reproductive development (Fig. 1D).

The response of *CsCSP2* to low temperature was assayed by exposing three contrasting cucumber ecotypes to 24 h of cold: the cultivated line ‘*9930*’, the semi-wild accession BN27, and W1983Hde—a mutant harboring a loss-of-function allele of *tfl1*. The results showed that *CsCSP2* gene expression was significantly elevated in all three lines after 24 h of cold exposure, indicating that low temperatures can induce *CsCSP2* expression in cucumbers (Fig. 1E). To further confirm that *CsCSP2* responds to cold stress, we performed GUS staining and GUS enzyme activity assays. These assays demonstrated that *CsCSP2* expression can indeed be induced by low temperatures (Fig. 1F, G).

We fused the CsCSP2 protein with YFP to generate the pHB-CsCSP2-YFP fusion construct. When transiently expressed in tobacco leaves, the fusion protein exhibited strong green fluorescence localized to the nuclei. These findings confirm that CsCSP2 is a nuclear protein (Figs. S2, S3). To further investigate the functional sites of CsCSP2 in cucumber, we performed RNA in situ hybridization to analyze its expression patterns and localization within wild-type cucumber tissues. The results revealed that *CsCSP2* is enriched in flower buds (FB) and the shoot apical meristem (SAM) (Fig. 1H). This spatial expression pattern resembles that of CsTFL1/CsTFL1d, key regulators of determinate growth in cucumber (Wen et al. 2021). Based on these findings, we propose that low temperature induces *CsCSP2* expression and that CsCSP2 regulates determinate growth by interacting with CsTFL1/CsTFL1d.

To investigate how CsCSP2 affects determinate growth in cucumbers through interaction with CsTFL1/CsTFL1d, we performed yeast two-hybrid, Bimolecular Fluorescence Complementation (BiFC), and Firefly Luciferase Complementation (LUC) assays. The yeast two-hybrid assay results showed that CsCSP2 interacts with CsTFL1/CsTFL1d in vitro (Fig. 1J). The interaction was further confirmed in the nucleus using a BiFC assay (Fig. 1I). Additionally, LUC assays demonstrated that CsCSP2 binds to CsTFL1/CsTFL1d in vivo (Fig. 1K). Together, these findings indicate that CsCSP2 physically interacts with CsTFL1/CsTFL1d both in vitro and in vivo. We also investigated whether CsCSP2 interacts with CsNOT2. However, yeast two-hybrid assays revealed no interaction between the two proteins (Fig. S4).

Using CRISPR/Cas9, we generated two independent knockout lines, *csp2#1* and *csp2#2*, in the ‘*S94*’ background (Fig. 1L). Both single mutants retained an indeterminate habit under early-spring conditions, but the *csp2/tfl1* double mutant displayed a mild determinate phenotype: the apical meristem was replaced by a floral meristem at a reduced node position (Fig. 1M, N). Consistently, qRT-PCR revealed highest expression of *CsAP1* and *CsLFY* in the *tfl1* single mutant, intermediate levels in the double mutant and lowest levels in csp2 single mutants and wild type (Fig. 1O,P). Previous studies indicate that *cstfl1* mutants exhibit significantly restricted growth under low-temperature, short-day conditions. Furthermore, the cucumber gene *CsTFL1d*, which is functionally similar to *CsTFL1*, regulates plant development and growth habit (Njogu et al. 2020; Wen et al. 2021). Based on these results, we propose that loss of CsCSP2 in the *tfl1* background appears to free CsTFL1d to bind CsNOT2a, thereby attenuating FT-mediated activation of flowering genes and partially mitigating the determinate phenotype of the csp2 tfl1 double mutant.

In this study, we demonstrate that CsCSP2 plays a pivotal role in mediating low temperature–induced determinate growth in cucumber, illuminating how thermal cues modulate the functional equilibrium between CsTFL1 and CsTFL1d (Fig. 1Q). Under low-temperature conditions, elevated CsCSP2 expression enables its binding to both CsTFL1 and CsTFL1d, thereby potentially disrupting the CsNOT2a-CsTFL1 complex. Prior work confirms direct interaction between CsNOT2a and CsTFL1/CsTFL1d. Although our assays did not reveal direct competition between CsCSP2 and CsNOT2a, the csp2/tfl1 double mutant’s phenotype—showing partial rescue of the determinate growth—supports a model in which CsCSP2 sequesters CsTFL1/CsTFL1d, attenuating the CsNOT2a-TFL1 interaction under low temperature.

Furthermore, in cultivated cucumber, domestication has led to sustained high expression of *FT*, resulting in FT protein accumulation under low-temperature environments and triggering terminal flower formation. Whether similar terminal flowering occurs in wild cucumbers, such as *C. sativus var. hardwickii*, remains unknown, limiting the extrapolation of our results beyond cultivated varieties. Hence, future investigations should examine whether wild cucumber species exhibit determinate growth phenotypes and dissect their molecular basis. Such efforts will not only deepen our understanding of cucumber domestication and thermal adaptation but may also uncover novel regulatory mechanisms by which environmental signals shape plant architectural diversity.

## Supplementary Information

Supplementary Material 1. Supplementary Materials and Methods.

Supplementary Material 2. Supplementary Figures.

Supplementary Material 3. Supplementary Table S1 and Table S2.

## Data Availability

The data that supports the findings of this study are available in the supplementary material of this article.
