# Supplementary material for: Low temperature-induced cold shock protein modulates determinate growth in cucumber
Source: Mol Hortic. 2026 Mar 1;6:16. doi: 10.1186/s43897-025-00199-3 (PMC12950231; doi:10.1186/s43897-025-00199-3)
Supplement: Supplementary file 1 — Supplementary Material 1. Supplementary Materials and Methods. [file 43897_2025_199_MOESM1_ESM.docx]

**Low Temperature-Induced Cold Shock Protein Modulates Determinate Growth in Cucumber**

Linghao Liu^a^, Haifan Wen^b^, Tiefeng Song^e^, Xiangyu Wang^f^, Junsong Pan^b^, Jian Pan^a, c*^, Tianlai Li ^a, c, d*^

^a^ College of Horticulture, Shenyang Agricultural University, Shenyang 110866, China

^b^ School of Agriculture and Biology, Shanghai Jiao Tong University, Shanghai, 200240, China

^c^ The Modern Facilities Horticultural Engineering Technology Center, Shenyang Agricultural University, Shenyang 110866, China

^d^ Key Laboratory of Protected Horticulture, Ministry of Education, Shenyang 110866, China

^e^ School of Life Sciences, Liaoning University, Shenyang, 110036, China

^f^ Liaoning Academy of Agricultural Sciences, Shenyang, 110161, China

^∗^Corresponding author: College of Horticulture, Shenyang Agricultural University, Shenyang 110866, China.

E-mail addresses: panjian@syau.edu.cn (J. Pan), tianlaili@126.com (T. Li).

**Materials and Methods**

**Plant materials and growth conditions**

The cucumber inbred lines, *S94*, *9930*, *BN27* and *W1983Hde* were used in this study. The cucumber seeds were germinated at 28 °C in dark and then grown in a growth chamber at 16 h day (25 °C) and 8 h night (18 °C) until two true-leaf stage. Seedlings of the mapping population were then transferred to greenhouse of Shenyang Agricultural University in early spring (February, the average temperature is 10-15 °C) and autumn (August, the average temperature is 25-35 °C). Water management and pest control were performed in accordance with standard protocols.

To examine the reaction of CsCSP2 and floral genes to temperature, *S94*, *9930*, *BN27* and *W1983Hde* plants were transferred to various artificial environments, including low temperature (early spring) (February, the average temperature is 10-15 °C) or normal temperature (autumn) (August, the average temperature is 25-35 °C), respectively. All treatment was conducted a minimum of three times, with no fewer than 20 seedlings per treatment. All materials were harvested and frozen immediately in liquid nitrogen and kept at -80 °C for RNA isolation.

**Histological examination**

Using digoxigenin-labeled RNA probes and applying them to paraffin-embedded tissue sections, the paraffin sectioning method follows Wen et al (Wen et al. 2021). Subsequent in situ hybridization experiments will be conducted, including pre-hybridization, hybridization, washing, color development, dehydration, and mounting. The results will be observed and recorded using a Nikon optical microscope. During the experiment, all reagents and consumables must be ensured to be free of RNase activity.The fixative should be pre-cooled at 4 °C and vacuumed on ice before use.

**Gene ontology (GO), KEGG Orthology (KO), and enrichment analysis**

GO seq R was used to perform GO enrichment analysis of DEGs. The enrichment of GO keywords among DEGs was deemed statistically significant if the corrected p-value was less than 0.05. The assignment of annotations from the Kyoto Encyclopedia of Genes and Genomes Pathway (KEGG; http://www.genome.jp/kegg) was conducted based on the KEGG database. The KOBAS software (Peking University, Beijing, China) was utilized to evaluate the enrichment of differentially expressed genes (DEGs) inside the KEGG pathways. The data used for heatmap are listed in Table S1.

**Bioinformatic analysis**

The phylogenetic tree was generated using MEGA 7.0 (http://www.megasoftware.net/

mega.php) and FigTree (http://tree.bio.ed.ac.uk/software/figtree/) by ClustalW alignment and the greatest likelihood approach. Bootstrap analysis was set to 1000 replicates, and other parameters were set to default. Bootstrap analysis was set to 1000 replicates, and other parameters were set to default. All genes utilized for the phylogenetic analysis are enumerated in Table S2.

**Generation of Transgenic cucumber Plants**

To generate the *csp2* mutants, two specific sgRNA target sites were designed and was generated in the ‘*S94*’ background using CRISPR/Cas9 gene editing technology. The resultant vectors were validated by sequencing, thereafter injected into *Agrobacterium tumefaciens* strain GV3101, and ultimately transformed into the cotyledon explants of the cucumber cultivar *S94*. The *tfl1* and *csp2*/*tfl1* mutants was kindly provided by Dr. Haifan Wen from Shanghai Jiao Tong University. Kanamycin-resistant transformants were identified and confirmed using PCR and DNA sequencing in the T0 and T1 generations. The primers utilized for vector construction are enumerated in Supplementary Table S3.

**Expression of CsCSP2 and floral genes**

To gain further insight into the relationship between environmental stimuli and the varieties of determinate growth in CsCSP2 plants, we planted 20 seedlings each of *csp2#1*, *csp2#2*, *csp2*/*tfl1*, *tfl1*, and *9930* in early spring (February, the average temperature is 10-15 °C) and autumn (August, the average temperature is 25-35 °C) of 2024, subsequently collected 10 shoot apices for gene expression assays 4 weeks later. The remaining 10 plants were allowed to grow for phenotypic observation. Wild-type individuals *S94* were utilized as controls for phenotypic observation and sample collection.

Cucumber plants were planted in early spring (February, the average temperature is 10-15 °C), and shoot apexes were harvested for total RNA extraction and cDNA synthesis, respectively. The CsACTIN2 (Csa6M484600.1) and CsUBI (Csa5M600925.1) were used as internal references. The mRNA relative expression level was calculated using the 2 ^-ΔΔCt^ method. Each test was performed separately with three biological replicates. The primer sequences were listed in Table S3.

**Subcellular localization**

The coding sequence (CDS) of CsCSP2, remove the termination codon, was integrated into the pHB-YFP reporter expression vector, which comprises the CaMV 35S promoter and YFP. The recombinant plasmid pHB-CsCSP2-YFP was effectively incorporated into the bacterial strain *Agrobacterium tumefaciens* GV3101. After infiltrating *Agrobacterium* into tobacco leaves, the leaves were carefully tended for one day under darkness, and then three days under optimal conditions (24 °C and 60 % relative humidity) with a photoperiod of 16 h of light and 8 h of darkness.

Thereafter, the epidermal cells of the transformed tobacco leaves were examined using a laser scanning confocal microscope (Leica, Germany). All experiments were conducted using three replicates. The primer sequences were listed in Table S3.

**Yeast two hybrid assay (Y2H)**

The complete coding sequence of CsCSP2 and CsNOT2a was integrated into the pGADT7 vector, whereas the complete coding sequences of CsTFL1, CsTFL1d and CsCSP2 were put into the pGBKT7 vector. The resultant plasmids were incorporated into the yeast strain AH109. The specific operation process is conducted according to the previous methods (Zhang et al., 2019). The combination of TRY-AD and AN1-BD functioned as a positive control. Table S3 presents the primer sequences employed in construction of plasmids for the yeast two-hybrid experiment.

**Bimolecular fluorescence complementation (BiFC) assay**

The complete coding sequences of CsCSP2 and CsTFL1/CsTFL1d, remove the stop codons, were amplified by PCR employing gene-specific primers. The sequences were subsequently integrated into the pXY106 and pXY104 vectors, respectively, which encompass the N or C terminus of YFP, so facilitating the formation of in-frame fusion proteins. Significantly, these vectors encompass the N or C terminus of YFP (Wen et al., 2021). Thereafter, the resultant plasmids were introduced into the *Agrobacterium tumefaciens* strain EHA105, which were subsequently infiltrated into *Nicotiana benthamiana* leaves to verify protein interactions. YFP fluorescence was observed with a confocal laser scanning microscope (TCS SP8; Leica). For the observation of yellow fluorescence, the excitation wavelength was established at 488 nm, with emission wavelengths ranging from 520 to 540 nm. Table S3 presents the primer sequences employed in construction of plasmids for the Bimolecular fluorescence complementation (BiFC) experiment.

**Firefly Luciferase Complementation (LUC) assay**

The full-length coding sequence of CsCSP2 was inserted into the pCAMBIA1300-cLUC vector, while the full-length coding sequences of CsTFL1/CsTFL1d were incorporated into the pCAMBIA1300-nLUC vector. The resultant plasmids were introduced into the *Agrobacterium tumefaciens* strain EHA105, which was then utilized to infiltrate the leaves of *Nicotiana benthamiana*. The resulting plasmids were then introduced into the Agrobacterium tumefaciens strain EHA105, which was subsequently used to infiltrate Nicotiana benthamiana leaves. Following a 3-day period, a 0.2 mmol·L^-1^ fluorescein solution (Promega; Madison, WI, USA) was applied to the surface of tobacco leaves, and the reaction was detected 30 minutes after the reaction using an imaging equipment (Night SHADE LB 985). Table S3 presents the primer sequences employed in construction of plasmids for the Firefly Luciferase Complementation (LUC) experiment.

**Statistical analysis**

All data were repeated at least three times. The statistical analyses were performed using SPSS Statistics (www.ibm.com/products/spss-statistics) software via Student’ s t-test. Significant differences were considered significant with a probability level of p < 0.05.

**References**

Jiang GX, Zeng J, Li ZW, Song YB, Yan HL, He JX, Jiang YM, Duan XW. Redox regulation of the NOR transcription factor is involved in the regulation of fruit ripening in tomato. *Plant Physiol*. 2020; 183: 671-685.

Wen HF, Pan J, Chen Y, Chen GQ, Du H, Zhang LY, Zhang KY, He HL, Wang G, Cai R, Pan JS. TERMINAL FLOWER 1 and TERMINAL FLOWER 1d respond to temperature and photoperiod signals to inhibit determinate growth in cucumber. Plant, Cell & Environment. 2021, 44(8):2580-2592.

Zhang LY, Pan J, Wang G, Du H, He HL, Pan JS, Cai R. Cucumber CsTRY negatively regulates anthocyanin biosynthesis and trichome formation when expressed in Tobacco. Frontiers in Plant Science, 10, 1232.
