# Supplementary material for: Low temperature-induced cold shock protein modulates determinate growth in cucumber
Source: Mol Hortic. 2026 Mar 1;6:16. doi: 10.1186/s43897-025-00199-3 (PMC12950231; doi:10.1186/s43897-025-00199-3)
Supplement: Supplementary file 2 — Supplementary Material 2. Supplementary Figures. [file 43897_2025_199_MOESM2_ESM.docx]

**Low Temperature-Induced Cold Shock Protein Modulates Determinate Growth in Cucumber**

Linghao Liu^a^, Haifan Wen^b^, Tiefeng Song^e^, Xiangyu Wang^f^, Junsong Pan^b^, Jian Pan^a, c*^, Tianlai Li ^a, c, d*^

^a^ College of Horticulture, Shenyang Agricultural University, Shenyang 110866, China

^b^ School of Agriculture and Biology, Shanghai Jiao Tong University, Shanghai, 200240, China

^c^ The Modern Facilities Horticultural Engineering Technology Center, Shenyang Agricultural University, Shenyang 110866, China

^d^ Key Laboratory of Protected Horticulture, Ministry of Education, Shenyang 110866, China

^e^ School of Life Sciences, Liaoning University, Shenyang, 110036, China

^f^ Liaoning Academy of Agricultural Sciences, Shenyang, 110161, China

^∗^Corresponding author: College of Horticulture, Shenyang Agricultural University, Shenyang 110866, China.

E-mail addresses: panjian@syau.edu.cn (J. Pan), tianlaili@126.com (T. Li).


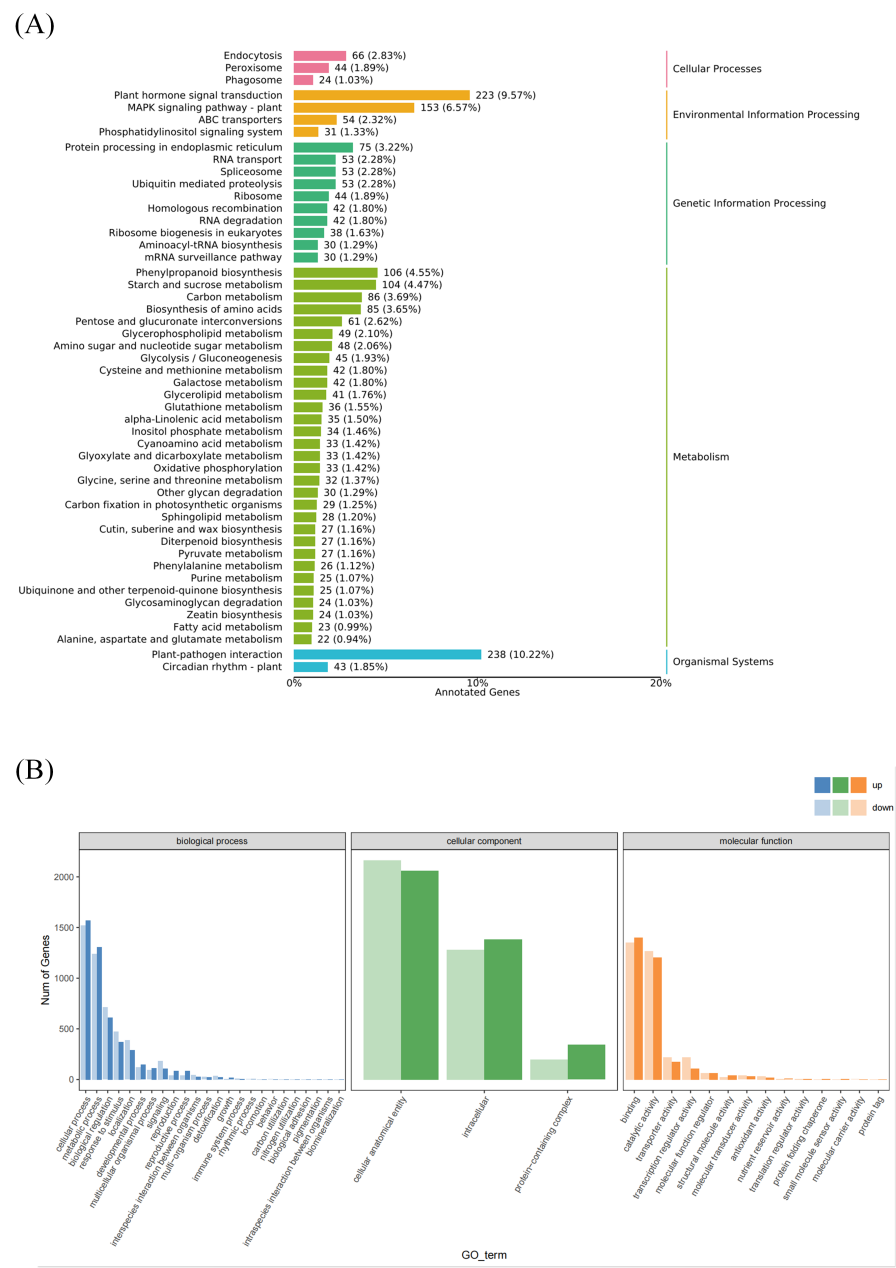


**Fig. S1** KEGG and GO enrichment analysis of all DEGs. (A) KEGG analysis of DEGs in early spring (February)-planted and autumn-planted cucumbers. (B) GO analysis of DEGs in early spring (February)-planted and autumn-planted cucumbers.


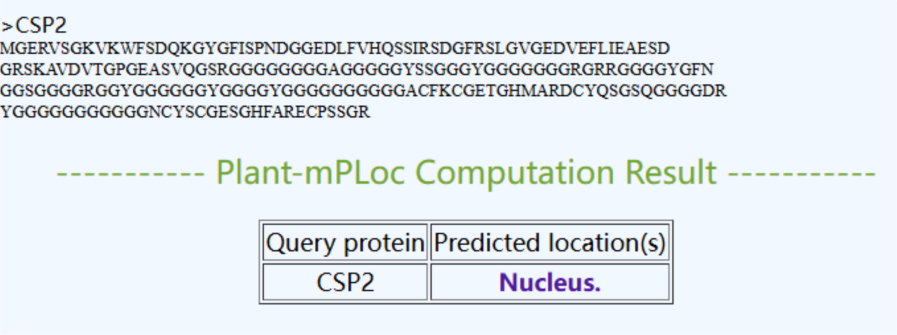


**Fig. S2** Prediction of subcellular localization of CsCSP2 proteins.


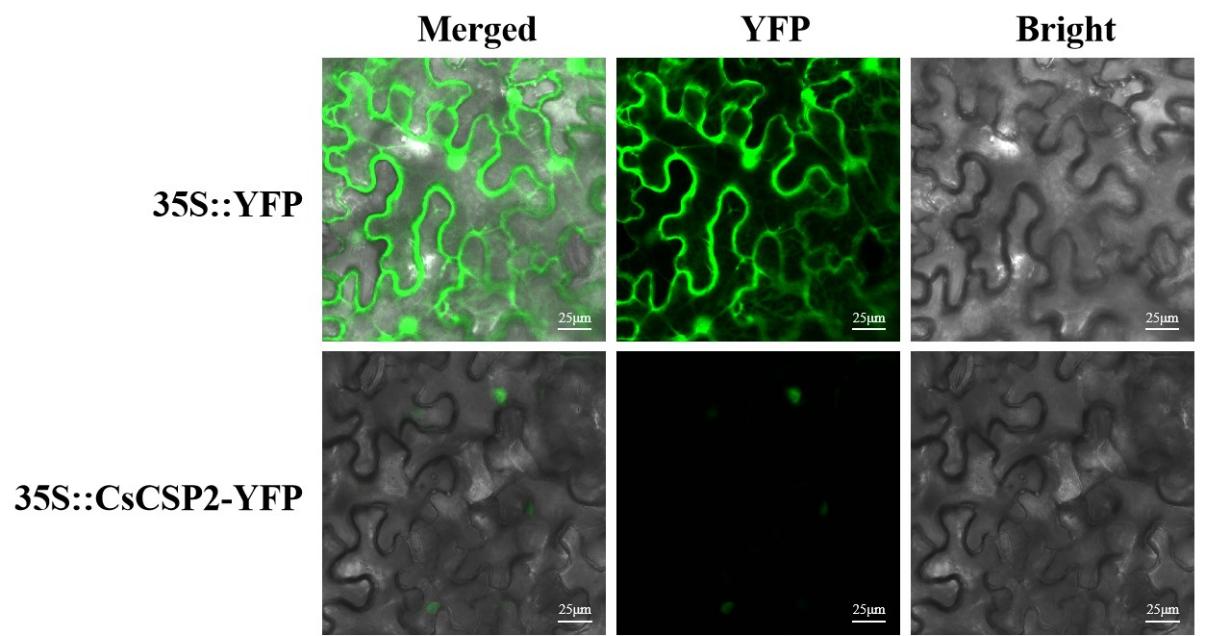


**Fig. S3** Subcellular localization of CsCSP2. Subcellular localization of CsCSP2-YFP fusion proteins in tobacco leaves.


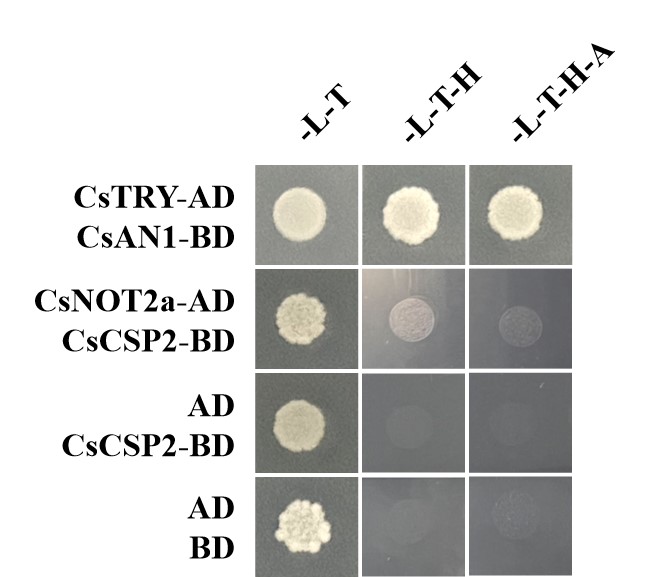


**Fig. S4** Y2H assay revealing the interaction between CsNOT2a and CsCSP2. Transformed yeast cells were grown on SD/-Trp/-Leu, SD/-Trp/-Leu/-His medium and SD/-Trp/-Leu/-His/-Ade medium.
